# Perceived Physical Literacy Levels in Spanish Adolescents: Differences Between Sexes and Age Groups

**DOI:** 10.3390/children12081017

**Published:** 2025-08-01

**Authors:** Raquel Albéniz-Pérez, Daniel Castillo, Pedro Duarte-Mendes, Javier Raya-González

**Affiliations:** 1 Department of Didactics of Musical, Plastic and Corporal Expression, Faculty of Education, University of Valladolid, 42004 Soria, Spain; raquel.albeniz@uva.es; 2 Valoración del Rendimiento Deportivo, Actividad Física y Salud y Lesiones Deportivas (REDAFLED), Faculty of Education, University of Valladolid, 42004 Soria, Spain; 3 Department of Sports and Well-Being, Polytechnic University of Castelo Branco, 6000-084 Castelo Branco, Portugal; pedromendes@ipcb.pt; 4 Sport Physical Activity and Health Research & Innovation Center (SPRINT), 6000-084 Castelo Branco, Portugal; 5 Research Group on Sport and Physical Education for Personal and Social Development (GIDEPSO), Department of Specific Didactics, Faculty of Education Sciences and Psychology, University of Córdoba, 14071 Córdoba, Spain; rayagonzalezjavier@gmail.com

**Keywords:** education, physical activity, health, exercise, sex

## Abstract

**Background/Objectives**: Perceived physical literacy (PPL) appears to be a relevant strategy for combating the prevalent sedentary lifestyle among young people. Therefore, understanding their PPL levels will facilitate the implementation of appropriate strategies for this purpose. Therefore, this study aimed to analyze the differences in PPL considering the sex dimension (i.e., males and females) and the age-group (i.e., early compulsory secondary education, late compulsory secondary education and baccalaureate). **Methods**: Seven-hundred-and-four Spanish students (age = 14.3 ± 1.6 years old) belonging to three different Spanish secondary schools voluntarily participated in this study. To assess adolescents’ perceptions of their physical literacy, the Spanish Adolescents’ Perceived Physical Literacy Assessment (S-PPLI) was used. This instrument consists of nine items equally distributed across three categories: self-perception and self-confidence, self-expression and communication with others, and knowledge and understanding. **Results**: Males obtained higher scores in all the indicators of PPL, except for items 1, 8 and 9, compared to their female counterparts (*p* < 0.05), while the oldest age-group reported higher scores in the indicators of knowledge and understanding category compared to students in the early years of compulsory secondary education (*p* < 0.01). **Conclusions**: Programs based on increasing the PPL should be implemented specifically for females. Also, similar programs must be included into scholar curriculums from the beginning of secondary education, with the aim of promoting improvements in the health and physical condition of Spanish adolescents.

## 1. Introduction

Perceived physical literacy (PPL) has emerged as a key multidimensional construct for promoting active and healthy lifestyles, particularly during childhood and adolescence [1,2]. Contemporary consensus defines this phenomenon as the combination of motor skills, knowledge, attitudes, motivation and confidence necessary to participate in physical activities across the lifespan, highlighting that PPL is not only related to physical competence, but also to emotional and social well-being [3]. Adolescence represents a pivotal developmental juncture, characterized by profound physiological and psychological transitions toward adulthood, and the progressive decline in physical activity levels [4]. Concretely, PPL was significantly associated with physical fitness, body composition, and physical activity levels among secondary school adolescents aged 14–18 [5]. Thus, understanding the PPL level is essential to design effective strategies to mitigate a sedentary lifestyle and its consequences, such as increased obesity, metabolic disorders and mental health problems [2]. For this purpose, it is essential to have adequate assessment instruments that allow decisions to be made based on robust results. Regarding this, [6] tried to validate of the Spanish Perceived Physical Literacy Instrument (S-PPLI) questionnaire in Spanish adolescents, based on an original questionnaire designed and validated for physical education teachers [7]. The authors proved that the S-PPLI is a valid and reliable measure of physical literacy among Spanish adolescents, after the conduction of several statistical analyses (e.g., confirmatory factor analysis, analyses for convergent validity or intraclass correlation coefficient) with positive results. These results suggest that the S-PPLI is an adequate instrument to assess physical literacy in Spanish adolescents, so it is used, due to the necessity of evaluating this construct and to design and implement significative strategies in this population.

Prior studies in several countries [8,9,10,11] have revealed consistent gender-based disparities. In particular, it has been reported that males demonstrated significantly higher self-perceived athletic competence, whereas females reported lower confidence in physical activity contexts [9,11]. Likewise, social class also appears to be another determining factor influencing PPL, given that adolescents with lower economic incomes and from ethnic minorities in the United States tended to report higher levels of physical inactivity [8,10]. Concurrently, physiological factors substantially modulate PPL development. Elevated body mass index (BMI) consistently correlates negatively with PPL measures [12], potentially reflecting both biomechanical constraints and weight-related stigmatization. Furthermore, the biological transitions of adolescence interact complexly with academic pressures and psychosocial changes to influence physical literacy trajectories [10,11]. In this sense, Rojo-Ramos et al. [11] found a significant negative correlation between advancing adolescent age and motivation for physical activity, suggesting developmental windows where targeted interventions may prove most effective. Despite these findings, these patterns derive primarily from non-Spanish populations, with limited examination of how educational transitions (e.g., between compulsory and post-compulsory schooling) influence PPL trajectories.

Multiple contextual factors appear to modulate PPL levels in adolescents, including the prevailing prioritization of academic achievement over holistic development, persistent gender disparities in sports participation, and significant variability in the quality of school-based physical education programs [12,13]. A key contextual factor is the age, mainly during adolescence, in which different developmental stages of age take place, which suggests the need to analyze PPL based on age groups (i.e., early compulsory secondary education, late compulsory secondary education and baccalaureate). Critically, PPL currently lacks explicit inclusion as a curricular competency within mandatory educational frameworks, resulting in inconsistent pedagogical attention across primary and secondary institutions [2]. This institutional gap provides a plausible explanation for the low PPL levels documented among Spanish adolescents [2], a concern further compounded by contemporary sedentary trends. This factor, together with the well-documented increase in sedentary behaviors—such as prolonged screen time, great social media consumption and reduced daily physical activity—has been linked to lower PPL, especially among adolescents [14].

Considering the aforementioned literature and the importance of knowing the PPL in adolescents, the main aim of this study is to analyze the differences in PPL considering the sex dimension (i.e., males and females) and the age-group (i.e., early compulsory secondary education, late compulsory secondary education and baccalaureate). Based on prior studies [9,11], we hypothesized that males would present higher levels of PPL than females, and that the oldest age-group would be better at self-management and self-confidence, while the youngest age-group would present the greatest values related to the enjoyment of physical activity.

## 2. Materials and Methods

### 2.1. Design

A descriptive, comparative and correlational design was implemented to deepen the knowledge of the level of PPL among Spanish adolescents by comparing between sexes and between age groups. One questionnaire of perceived physical literacy, which was previously validated in Spanish adolescents [6], was administered to the participants following the standardized procedures. In the same session, participants were asked to complete their sex and age, to be classified according to this. Questionnaires were completed using computers and mobile devices in the classroom with the supervision of the teacher for solving some questions, and lasted between 10 and 15 min.

### 2.2. Participants

Seven hundred and four Spanish students (age: 16.3 ± 1.6 years, body mass: 55.1 ± 11.7 kg, stature: 164.7 ± 10.4 cm) belonging to 3 different secondary schools participated in this study during the academic year 2024/2025. Schools were selected through a convenience sampling approach based on institutional accessibility and willingness to participate in the study. These schools were allocated in Andalucia, Madrid and Galicia regions, and all of them were public schools, with the students and their families being of a medium socioeconomic level. Three hundred and seventy-four participants were males (53.13%) and three hundred and thirty were females (46.87%). Regarding the classification by age-group, 255 students (36.22%) belonged to early compulsory secondary education, 265 students (37.64%) to late compulsory secondary education and 184 students (26.14%) to baccalaureate. To be included in the study, the following eligibility criteria had to be met: (1) be between 12 and 18 years of age, (2) accept to participate in the study, (3) have obtained the consent of parents/guardians, (4) have residency in Spain, and (5) have not presented pathologies preventing the practice of physical activity.

### 2.3. Ethics

The research received the approval of the Ethics Committee of the University of Córdoba (code: CEIH-24-47) and followed the ethical principles of the Declaration of Helsinki by the 64th General Assembly of the World Medical Association (Fortaleza, Brazil, 2013) and in compliance with Law 14/2007 on Biomedical Research.

### 2.4. Perceived Physical Literacy (PPL)

The Spanish Adolescents’ Perceived Physical Literacy Assessment (S-PPLI) was used to assess adolescents’ perceptions of their physical literacy. Data collection was conducted in person during physical education classes during the academic year 2024/2025. While the original S-PPLI consisted of 18 items, this study used the validated 9-item short version proposed by López-Gil et al. [6]. The abbreviated version was developed through confirmatory factor analysis and internal consistency testing, and demonstrated acceptable psychometric properties, including good reliability and construct validity (values from 0.53 to 0.77). The selection of the 9 items was based on statistical performance (e.g., factor loadings and item-total correlations) while maintaining theoretical coherence with the original construct. The final version retains a balanced structure, with three items representing each of the following subscales: (1) self-perception and self-confidence, (2) self-expression and communication with others, and (3) knowledge and understanding, as shown in Table 1. Each item was rated on a 5-point Likert scale ranging from 1 (totally disagree) to 5 (totally agree). The total PPL score was calculated as the sum of all item scores. In the present study, the internal consistency of the instrument was good (Cronbach’s alpha = 0.861), even when each subscale was analyzed in isolation (subscale 1 = 0.778; subscale 2 = 0.613; subscale 3 = 0.754).

### 2.5. Statistical Analysis

Data are presented as mean ± standard deviation (SD). The normal distribution of the data and the homogeneity of variances were tested using the Shapiro–Wilk and Levene test, and statistical parametric techniques were performed. A Confirmatory Factor Analysis (CFA) was performed to cross-validate and confirm the three-factor structure derived in the analysis. Pearson’s product-moment correlation coefficient (r) with a 95% confidence interval (CI) was used to examine the relationship between PPL domains. The following scale of magnitudes was used to interpret the correlation coefficients: <0.1, trivial; 0.1–0.3, small; 0.3–0.5, moderate; 0.5–0.7, large; 0.7–0.9, very large; and >0.9, nearly perfect [15]. The t-test for independent samples was performed to evaluate the differences according to sex (i.e., males and females). A one-way analysis of variance (ANOVA) with a least-significant-difference post hoc comparison (Bonferroni correction) was used to examine mean differences among age-groups (i.e., early compulsory secondary education, late compulsory secondary education and baccalaureate). A multivariate analysis of variance (MANOVA) was applied to simultaneously examine sex and age-group effects across the three PPL subscales. Mean differences were calculated in percentage (%Diff.) by the following formula: %Diff. = ((mean 1 − mean 2)/mean 2) × 100. Practical significance was assessed by calculating Cohen’s effect size (ES) [16]. ES of above 0.8, between 0.8 and 0.5, between 0.5 and 0.2, and lower than 0.2 were considered large, moderate, small and trivial, respectively. Data analysis was carried out using the JASP 0.16.3.0 software (University of Amsterdam, Amsterdam, The Netherlands). Statistical significance was established at *p* < 0.05.

## 3. Results

**Figure 1:**
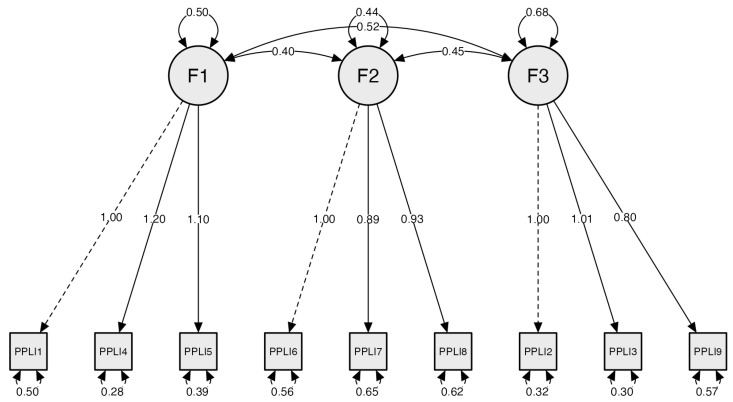
Factor loading of the three-factor model structure for the S-PPLI questionnaire.

The CFA showed the following values: chi square (*x*^2^ = 72.800; *df* = 24; *p* < 0.01; CFI = 0.990; *RMSEA* = 0.056; and *SRMR* = 0.031), revealing that the three-factor validity of physical literacy was satisfactory (Figure 1).

**Figure 2:**
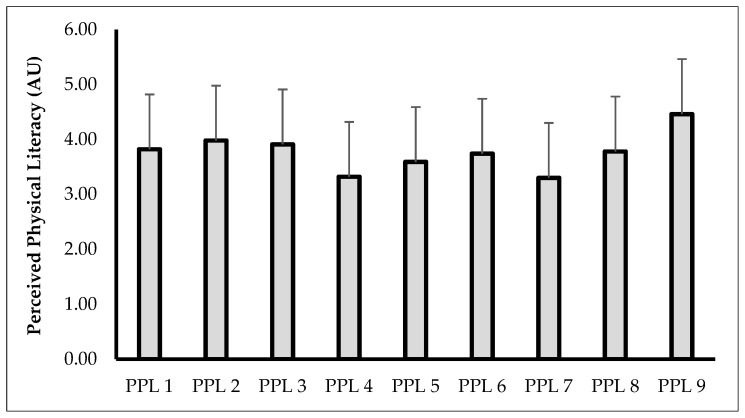
Descriptive data of perceived physical literacy (PPL) in Spanish students.

Figure 2 displays the descriptive data of each domain of PPLI in Spanish adolescent students showing scores between 3.30 ± 1.16 AU in PPL7 and 4.46 ± 0.83 AU in PPL9. The total score recorded by the 704 students in PPLI was 33.89 ± 6.52 AU. All items (from PL1 to PL9) of S-PPLI demonstrated a significant correlation (*p* < 0.001, *r* = 0.212–0.633 [small to moderate effects]).

Table 2 shows the comparisons between males and females in each item and PPLtotal of S-PPLI. Males obtained higher scores from PPL2 to PPL7 (*p* < 0.001–0.027, ES = 0.17–0.67 [trivial to moderate effect]) and in PPLtotal (*p* < 0.001, ES = 0.38 [small effect]) in comparison to females, significantly. Otherwise, no significant differences (*p* > 0.05) were found in PPL1, PPL8 and PPL9 between Spanish males and females. Regarding to the analysis of the three subscales of the questionnaire, higher values were found in all of them in favor of male participants (*p* = 0.002 – <0.001; ES = 0.23–0.51 [small-moderate effect]).

The comparisons according to each group (i.e., early compulsory secondary education, late compulsory secondary education and baccalaureate) in each item and PPLtotal of S-PPLI are presented in Table 3. Baccalaureate students reported higher scores in PPL8 and PPL9 in comparison to early compulsory secondary education students (*p* = 0.003–0.007, ES = 0.29 [small effect]) and higher PPL8 score than late compulsory secondary education students (*p* = 0.004, ES = 0.31 [small effect]). Nevertheless, no significant differences (*p* > 0.05) were found from PPL1 to PPL7 between baccalaureate students and the other two age-groups. On the other side, no significant differences (*p* > 0.05) were reported in all PPL domains between early compulsory secondary education and late compulsory secondary education. No between-age groups differences were found attending to the analysis of the three subscales of the questionnaire (*p* > 0.05).

In addition, the MANOVA revealed no significant interaction effect between sex and age group on PPL subscales (*p* = 0.433).

## 4. Discussion

Understanding PPL could be a key strategy of compulsory secondary education to improve adolescents’ physical activity levels, and thus to foster an active and healthy lifestyle that helps to reduce health problems in adulthood. In this regard, the main aim of this study was to analyze the differences in PPL considering the sex dimension and the age group among Spanish adolescents. This is the first study to examine PPL in Spanish adolescents, differentiation between three different age groups, which provides highly valuable information that covers a large part of adolescence, and its changes related to PPL. The main findings showed that males obtained higher scores in some indicators of PPL compared to females, and the oldest age-group reported higher scores in the indicators of knowledge and understanding subscale compared to students in the early years of compulsory secondary education.

To advance understanding of adolescents’ PPL, analysis must extend beyond aggregate scores to examine individual categories comprised by the S-PPLI questionnaire. When considering the entire cohort, mean item scores consistently exceeded the scale midpoint, ranging from 3.30 ± 1.16 AU (Item 7: *I understand how physical activity benefits my health*) to 4.46 ± 0.83 AU (Item 1: *I feel confident when trying new physical activities*), with an overall mean of 3.77 ± 0.35 AU. The cumulative S-PPLI score of 33.89 ± 6.52 AU further reflects moderate-to-high PPL perceptions, a finding that contrasts with reports of low physical literacy in comparable populations [17]. This controversy may partly reflect methodological differences, as the cited study employed distinct assessment tools. Crucially, these results underscore the need for a more comprehensive investigation about the topic (i.e., sex and age-group differences) to inform targeted educational strategies. Also, these results could be influenced by the specific socioeconomic characteristics of the participants (i.e., medium level), who, in general, have access to different extracurricular activities related to physical activity and sport, which could be limited in low socioeconomic contexts.

Traditionally, it has been observed that females tend to have lower levels of physical fitness and physical activity compared to males [18,19]. Therefore, it seems necessary to compare their levels of PPL, as this could provide relevant evidence to help reduce these inequalities [2]. In our study, males scored significantly higher than females on items PPL2 to PPL7 and on the total PPL score. Also, these differences were found in the three subscales. In this way, males showed higher values in self-perception, self-confidence, self-expression and communication with others, and knowledge and understanding. While statistically significant, these findings warrant cautious interpretation given their consistently trivial-to-moderate effect sizes, suggesting practical applications would require holistic, sustained interventions rather than isolated adjustments. However, no significant differences were found in PPL9 —I am aware of the health-related benefits of sports. The absence of gender differences in this item carries significant pedagogical implications. While gendered disparities persist in motivational and behavioral domains [9,11], this finding suggests that school-based health education, when delivered through standardized curricula and evidence-based public health messaging, can effectively transcend sociocultural gender barriers to knowledge acquisition. This aligns with competency-focused pedagogical models in Spain’s educational framework, where health literacy constitutes a cross-cutting objective in secondary education. The finding suggests that cognitive outcomes in health education are more equitably achievable than attitudinal or behavioral outcomes, highlighting structured pedagogy’s potential to mitigate gender gaps in Science, Technology, Engineering, Mathematics (STEM)-related health knowledge. Along these lines, other authors have also found that males aged 8 to 12 report higher total PPL and greater life satisfaction than females [12], indicating that such findings are also present at lower educational levels. Despite geographical diversity among participating high schools, these results suggest that Spain’s standardized public school system and national policies drive consistent outcomes. Nevertheless, Tremblay et al. [17] did not find such sex-based differences in PPLtotal in a study involving 10,034 Canadian participants aged 8 to 12. These discrepancies could be partially explained by cultural factors and the specific sample involved, such as prevailing gender norms, traditional views on physical activity, or educational priorities in Spanish society. In the current Spanish context, for instance, physical activity may still be influenced by gendered expectations or limited by institutional constraints within the school system. However, due to the scarcity of specific empirical studies on these cultural dimensions, further research is needed to clarify their exact influence. Based on the aforementioned findings, it could be interesting to promote physical activity programs tailored to the interests of females, moving away from traditional sports that may be sexualized and/or carry an exclusive cultural weight. This would help girls feel more comfortable and confident when engaging in physical activity, enabling them to more strongly adhere to the physical activity and fitness practices proposed in school and extracurricular programs, thereby improving their healthy lifestyle habits and their overall relationship with physical activity. No between-gender differences were obtained when subscales were analyzed, suggesting that is necessary to perform item-by-item analyses in order not to overlook significant differences.

PPL analysis highlights how adolescents relate to physical activity and healthy lifestyle habits, which may have a direct influence on their behaviors and routines in adulthood. [19]. The ENERGYCO study [20] associated high levels of PPL not only with greater physical activity, but also with better sleep patterns and less screen time. In the present study, age was found to be an influential factor in adolescents’ PL, as Baccalaureate students reported higher scores in PPL8 and PPL9 compared to early compulsory secondary education students, and higher PPL8 scores than late compulsory secondary education students. In this regard, it could be that older students are more mature and better able to cope with challenges and identify the benefits of engaging in physical activity [21]. Also, in the second year of Baccalaureate, Physical Education lessons are voluntary, so it is possible that most interested students have chosen this, related to higher values of PPL. Likewise, future studies could incorporate differential item functioning analyses to ensure the measure’s reliability and validity across diverse adolescent age groups. On the other hand, no significant differences were found in the PL domains between early and late compulsory secondary education students. These results may be explained by the standardized curricula possibly not matching advancing neuromotor skills, while the shift from skill acquisition to specialization and peer conformity can stabilize perceived competencies. Therefore, it would be advisable to include learning situations in the physical education curriculum [22] that enable younger students to effectively resolve challenges and develop greater awareness of the benefits of physical activity. In addition, no significant differences were found when subscales were analyzed, suggesting the relevance of perform item-by-item analyses. Similarly, no significant differences were obtained when simultaneously examining gender and age group, reinforcing the initial idea of doing so in isolation, due to the marked and differentiated characteristics between male and female adolescent students, to avoid biasing the information and ignoring between-gender differences masked by the influence of age. Future research should adopt longitudinal designs to examine how PPL changes over time and investigate potential mediating factors that might explain the observed sex and age differences.

This study has several limitations. First, a more balanced distribution of participants across age groups and involving more than three public schools would have provided more accurate and generalizable data for comparison. Second, the absence of information on participants’ extracurricular physical activity levels, an important factor influencing adolescents’ PPL [13,14,20], limits the interpretation of results. Additionally, future research should adopt a longitudinal design to assess PPL domains throughout adolescence, enabling better alignment of both curricular and extracurricular physical activity programs with developmental needs. Third, we acknowledge that BMI and certain physical fitness profiles may be related to certain components of PPL [23], and future research could incorporate these data to more comprehensively understand the factors influencing physical literacy development. Finally, an important limitation is the homogeneity of the sample, composed exclusively of public-school students from three Spanish regions, predominantly representing middle-class socioeconomic status. Socioeconomic factors significantly influence both access to physical activity opportunities and the development of physical literacy competencies [24]; therefore, these findings should be interpreted with caution when considering their applicability to other populations. This limits the generalizability of the findings to students from private schools, other regions, or significantly different socioeconomic contexts. However, this focus provides a context-specific understanding of the reality within public schools in these regions under these prevailing conditions.

## 5. Conclusions

The findings of this study showed that males presented higher scores in PPL domains compared to females, highlighting better self-perception, self-confidence, self-expression and communication with others, and knowledge and understanding. In addition, while Baccalaureate students reported higher scores in knowledge and understanding compared to early and late compulsory secondary education students, there are no significant differences between early and late secondary education students. These results provided valuable insights for professionals in charge of designing and adjusting the educational curriculums in physical education, such as integrating more student-centered activities, promoting inclusive teaching strategies, or emphasizing physical literacy development based on students’ diverse needs and experiences. Future research could explore the long-term effects of school-based research interventions on PPL through longitudinal designs. In practical terms, given females’ lower physical literacy levels identified in this study, we recommend integrating gender-tailored physical activity programs into school curricula through three approaches: activity diversification by replacing traditional sports with non-competitive alternatives (e.g., dance, martial arts, adventure circuits) aligned with female adolescents’ interests; pedagogical adaptation through educator training to create psychologically safe environments using single-gender sessions, body-neutral language, and student-co-designed challenges; and curricular embedding by allocating approximately 30% of mandatory physical education time to these tailored modules, complemented by flexible after-school activity hubs where students select a non-traditional physical option. This framework moves beyond theoretical suggestions to provide implementable pathways for reducing gendered barriers while advancing physical literacy development.

## Figures and Tables

**Table 1:** Spanish perceived physical literacy instrument (S-PPLI) used in this study.

Subscale 1. Self-perception and self-confidence.	
PPL1	I am physically fit, according to my age.
PPL4	I have self-management skills for fitness.
PPL5	I have self-assessment skills for health.
Subscale 2. Self-expression and communication with others.	
PPL6	I have strong social skills.
PPL7	I have confidence in wild/natural survival.
PPL8	I am able to handle problems and difficulties.
Subscale 3. Knowledge and understanding.	
PPL2	I have a positive attitude and interest in sports.
PPL3	I value myself or others by doing sports.
PPL9	I am aware of the health-related benefits of sports.

Note. PPL: perceived physical literacy.

**Table 2:** Spanish perceived physical literacy instrument (S-PPLI) according to sex in Spanish students.

Domains	Males		Females				
	Mean	SD	Mean	SD	%Diff.	*p*	ES
PPL1	3.85	1.09	3.79	1.02	1.69	0.413	0.06
PPL2	4.29	0.98	3.62	1.19	15.38	<0.001	0.67
PPL 3	4.11	0.99	3.68	1.09	10.53	<0.001	0.44
Subscale 1	11.04	2.78	10.40	2.55	5.80	0.002	0.23
PPL 4	3.48	1.15	3.15	1.16	9.44	<0.001	0.29
PPL 5	3.71	0.99	3.46	0.99	6.60	0.001	0.25
PPL 6	3.82	1.02	3.65	1.08	4.60	0.027	0.17
Subscale 2	11.12	2.34	10.49	2.40	5.67	<0.001	0.27
PPL 7	3.52	1.12	3.06	1.16	13.23	<0.001	0.42
PPL 8	3.78	0.95	3.79	0.98	0.18	0.926	0.01
PPL 9	4.50	0.84	4.40	0.81	2.29	0.100	0.12
Subscale 3	12.90	2.34	11.71	2.51	9.22	<0.001	0.51
PPLtotal	35.05	6.45	32.57	6.35	7.07	<0.001	0.38

Note. PPL: perceived physical literacy; SD: standard of deviation; %Diff.: mean differences in percentage; *p*: level of significance; ES: effect size.

**Table 3:** Spanish perceived physical literacy instrument (S-PPLI) according to age-group in Spanish students.

Domains	Early Compulsory Secondary Education	Late Compulsory Secondary Education	Baccalaureate	Early vs. Late (%Diff.; *p*; ES)	Early vs. Baccalaureate (%Diff.; *p*; ES)	Late vs. Baccalaureate (%Diff.; *p*; ES)
PPL 1	3.79 ± 1.13	3.85 ± 1.06	3.82 ± 0.94	1.61; 1.000; 0.05	0.87; 1.000; 0.03	−0.73; 1.000; 0.03
PPL 2	4.01 ± 1.16	3.96 ± 1.14	3.97 ± 1.09	1.32; 1.000; 0.11	1.02; 1.000; 0.04	0.30; 1.000; 0.01
PPL 3	3.94 ± 1.12	3.85 ± 1.06	3.95 ± 0.99	2.23; 1.000; 0.08	0.13; 1.000; 0.09	2.36; 1.000; 0.09
Subscale 1	10.62 ± 2.72	10.78 ± 2.78	10.84 ± 2,52	1.48; 1.000; 0.06	2.03; 1.000; 0.09	0.55; 1.000; 0.02
PPL 4	3.27 ± 1.15	3.35 ± 1.20	3.35 ± 1.14	2.39; 1.000; 0.07	2.32; 1.000; 0.07	0.06; 1.000; 0.01
PPL 5	3.56 ± 1.05	3.58 ± 0.97	3.66 ± 0.97	0.67; 1.000; 0.02	2.89; 0.816; 0.10	2.24; 1.000; 0.08
PPL 6	3.73 ± 1.12	3.69 ± 1.07	3.84 ± 0.91	1.23; 1.000; 0.04	2.94; 0.797; 0.10	4.14; 0.341; 0.15
Subscale 2	10.84 ± 2.43	10.67 ± 2.48	11.03 ± 2.20	6.70; 1.000; 0.07	1.72; 1.000; 0.09	3.26; 0.336; 0.16
PPL 7	3.40 ± 1.12	3.29 ± 1.22	3.20 ± 1.13	3.21; 0.850;0.10	5.89; 0.224; 0.18	2.77; 1.000; 0.07
PPL 8	3.71 ± 0.98	3.70 ± 0.97	4.00 ± 0.90	0.43; 1.000; 0.02	7.03; 0.007; 0.29	7.43; 0.004; 0.31
PPL 9	4.35 ± 0.91	4.45 ± 0.83	4.61 ± 0.66	2.16; 0.546; 0.11	5.74; 0.003; 0.29	3.66; 0.098; 0.20
Subscale 3	12.30 ± 2.65	12.25 ± 2.49	12.53 ± 2.25	0.41; 1.000; 0.02	1.84; 1.000; 0.10	2.23; 0.756; 0.12
PPLtotal	33.75 ± 6.78	33.68 ± 6.66	34.38 ± 5.92	0.23; 1.000; 0.01	1.81; 0.973; 0.09	2.04; 0.792; 0.11

Note. PPL: perceived physical literacy; SD: standard of deviation; %Diff.: mean differences in percentage; *p*: level of significance; ES: effect size.

## Data Availability

Data would be available after request to the corresponding author due to privacy reasons.
